# Potassium fertilizer promotes the thin-shelled Tartary buckwheat yield by delaying senescence and promoting grain filling

**DOI:** 10.3389/fpls.2024.1385548

**Published:** 2024-05-02

**Authors:** Lulu Tang, Jingang Tang, Kaifeng Huang, Xiaoyan Huang

**Affiliations:** ^1^School of Life Science, Guizhou Normal University, Guiyang, China; ^2^Guizhou Institute of Mountain Resources, Guizhou Academy of Sciences, Guiyang, China

**Keywords:** antioxidant enzyme activity, root morphological, rhizosphere soil nutrients, grain weight, thin-shelled Tartary buckwheat

## Abstract

The application rate of potassium fertilizer is closely related to the yield of crops. Thin-shelled Tartary buckwheat is a new variety of Tartary buckwheat with the advantages of thin shell and easy shelling. However, little is known about application rate of potassium fertilizer on the yield formation of thin-shelled Tartary buckwheat. This study aimed to clarify the effect of potassium fertilizer on the growth and yield of thin-shelled Tartary buckwheat. A field experiment to investigate the characteristics was conducted across two years using thin-shelled Tartary buckwheat (Miku 18) with four potassium fertilizer applications including 0 (no potassium fertilizer, CK), 15 (low-concentration potassium fertilizer, LK), 30 (medium-concentration potassium fertilizer, MK), and 45 kg·ha^−1^ (high-concentration potassium fertilizer, HK). The maximum and average grain filling rates; starch synthase activity; superoxide dismutase and peroxidase activities in leaves; root morphological indices and activities; available nitrogen, phosphorus, and organic matter content in rhizosphere soil; urease and alkaline phosphatase activities in rhizosphere soil; plant height, main stem node number, main stem branch number, leaf number; grain number per plant, grain weight per plant, and 100-grain weight increased first and then decreased with the increase in potassium fertilizer application rate and reached the maximum at MK treatment. The content of malondialdehyde was significantly lower in MK treatment than in other three treatments. The yields of thin-shelled Tartary buckwheat treated with LK, MK, and HK were 1.22, 1.37, and 1.07 times that of CK, respectively. In summary, an appropriate potassium fertilizer treatment (30kg·ha^−1^) can delay the senescence, promote the grain filling, and increase the grain weight and final yield of thin-shelled Tartary buckwheat. This treatment is recommended to be used in production to achieve high-yield cultivation of thin-shelled Tartary buckwheat.

## 1 Introduction

Buckwheat belongs to *Fagopyrum* Mill. of Polygonaceae. It has three main cultivated species that are widely planted in China: common buckwheat (*Fagopyrum esculentum* Moench), Tartary buckwheat (*Fagopyrum tataricum* Gaertn), and golden buckwheat (*Fagopyrum cymosum*) (Zhou et al., 2023a). Tartary buckwheat grain contains higher flavonoids, polyphenols, protein, fat, starch, dietary fiber, minerals, vitamins, and trace elements than wheat, rice, corn, and other crops (Xiang et al., 2022). It has the effects of preventing and treating hypertension, coronary heart disease, and diabetes; anti-cancer; and anti-tumor (Singh et al., 2021; Yao et al., 2022). Thus, it is considered to be the most medicinal and food homologous food crop with the strongest health care function, and it has high research and development value.

Almost all Tartary buckwheat varieties have the common problem that the grain shell is thick and difficult to shell in production (Chen, 2018). Thin-shelled Tartary buckwheat is a new variety of Tartary buckwheat bred by hybridization breeding in recent years. It has the advantages of no groove, thin shell, and easy shelling. It overcomes the scientific problem that Tartary buckwheat is difficult to shell, but its yield is low, which seriously affects its industrialization process. Therefore, clarifying the mechanism of yield formation of thin-shelled Tartary buckwheat and proposing cultivation measures to increase yield are urgent scientific problems that need to be solved to promote the development of the Tartary buckwheat industry.

The grain filling period is an important physiological stage of crop growth. During this period, the grain is enriched, and the grain filling process is closely related to the enrichment of the grain and the formation of the final yield (Zhang et al., 2023a). The starch in Tartary buckwheat grains accounts for about 70%–80%, so the filling process of these grains is actually the accumulation process of starch (Guo et al., 2022). Photosynthetic assimilates are transported from source organs (e.g., leaves and stems) to sink organs (grains) in the form of sucrose and form starch through a series of enzymatic reactions (Huang et al., 2020). In this process, the key enzymes, such as adenosine diphosphate glucose pyrophosphorylase (AGPase) and soluble starch synthase (SSS), of the sucrose–starch metabolic pathway in grains play an important role (Zhang et al., 2020). Therefore, these studies indicate that AGPase and SSS activities in grains are closely related to grain filling and yield formation.

Potassium, which is involved in various metabolic processes in plants, is one of the three major mineral nutrients necessary for plant growth and development (Broadley et al., 2004). Potassium is mainly distributed in the most active metabolic organs of plants. It plays a key role in photosynthesis, sugar synthesis, and resistance enhancement in plants. It also plays an important role in the formation of crop yield and quality (Jin et al., 2011; Wang et al., 2013). Gu et al. (2023) found that potassium fertilizer application can significantly increase the number of pods per plant and the number of seeds per pod, thereby increasing the grain yield of rape. Lei et al. (2015) found that an appropriate application of potassium fertilizer can significantly promote the grain filling of rice and ultimately achieve high yield. At present, in China’s agricultural production, farmers tend to apply nitrogen or phosphorus fertilizer to improve crop yield, ignoring potassium fertilizer. So, potassium deficiency has become one of the biggest limiting factors to improve crop yield. Influenced by the traditional concept that Tartary buckwheat is a barren-tolerant crop, this phenomenon is particularly obvious in the actual production of Tartary buckwheat, seriously affecting its yield (Huang et al., 2019). Wang et al. (2019) found that an appropriate potassium fertilizer treatment can promote the growth of roots, increase its absorption of rhizosphere soil nutrients, and then increase the final yield of Tartary buckwheat. These studies have shown that the application of potassium fertilizer is closely related to the yield of buckwheat. To date, studies with potassium fertilizer have focused on the yield of buckwheat only from the perspective of cultivation measures and have not analyzed the physiological characteristics. The current study hypothesized that a certain application of potassium fertilizer may increase the grain weight and yield of Tartary buckwheat by regulating grain filling and leaf senescence. However, studies relevant to this hypothesis are currently lacking, particularly related research on thin-shelled Tartary buckwheat Therefore, in the present work, a new variety of thin-shelled Tartary buckwheat named Miku 18 was used as the test material, and different potassium fertilizer applications were set up to study their effects on the grain filling characteristics, root morphology and physiology, senescence, and yield formation of thin-shelled Tartary buckwheat. The major objective was to reveal the effect of potassium fertilizer on the growth and yield of thin-shelled Tartary buckwheat. The results provide theoretical basis and technical reference for the high-yield cultivation of thin-shelled Tartary buckwheat.

## 2 Materials and methods

### 2.1 Plant materials and growth

The thin-shelled Tartary buckwheat cultivar Miku 18 was provided by the School of Life Science of Guizhou Normal University, China. The experiment was conducted during the growing season of Tartary buckwheat (August–November) from 2021 to 2022 at Xiaba’s Cultivation Experimental Station of Guizhou Normal University, Guiyang City, Guizhou Province, China (1250 m, 106.9493°E, 26.7309°N). The soil used was xanthic ferralsols, and the nutrient contents of the shallow tillage layer (0–20 cm) at the test site were as follows: 14.12 mg·kg^−1^ available nitrogen, 29.43 mg·kg^−1^ available phosphorus, 10.23mg·kg^−1^ available potassium, and 24.36‰ organic matter. The soil nutrient contents were determined using a multichannel intelligent soil nutrient meter (OK-V24, China).

The experiment was laid out in a randomized complete block design with three replications. The plot area was 2m×5m, and a 35cm ridge was built between the plots, wrapped and isolated with agricultural film (polyethylene, 0.06 mm thick) to prevent fertilizer mixing. On the basis of previous studies, four treatments of potassium fertilizer (potassium chloride, containing 60% K_2_O) were set up: 0K (CK, 0kg·ha^−1^), low-concentration potassium fertilizer (LK, 15kg·ha^−1^), medium-concentration potassium fertilizer (MK, 30kg·ha^−1^), and high-concentration potassium fertilizer (HK, 45kg·ha^−1^). Nitrogen fertilizer (urea, containing 46%N) and phosphorus fertilizer (calcium superphosphate, containing 14% P_2_O_5_) were applied at the best local dosages of 135 and 70kg·ha^−1^, respectively. The three fertilizers were mixed and applied as base fertilizer at one time, and no fertilizer was applied during the whole growth period. Sowing was carried out on August 16, 2021, and August 18, 2022. The row spacing and seeding rate were 33cm and 3.65g·m^−2^, respectively. The basic seedlings were maintained at 90–100 plants·m^−2^ (thinning or supplementing seedling at the seedling stage to maintain planting density). When 70% of the Tartary buckwheat grains in each plot matured, they were harvested (November 20, 2021, and November 21, 2022). During flowering and grain-filling, artificial irrigation was carried out in accordance with the principle of no less than 80% of field capacity, and the other periods depended on natural precipitation. The other field management and pest control were consistent with local high-yield cultivation.

### 2.2 Sample preparation

At initial anthesis, the plants with consistent growth and no pests and diseases were selected from each treatment plot, and about 2000 flowers (per plot, located on the top 4–6 nodes of the main stem) that boomed on the same day were marked on the calyx with a brush dipped in black ink. After 7 days, the marked flowers were sampled for the first time and every 7 days until maturation. In each treatment plot, 250 labeled grains were taken, of which 150 were divided into three parts, and the 100-grain weight was calculated after drying. The remaining 100 grains were frozen in liquid nitrogen for 30 s and stored in a refrigerator at−80°C.

At the seedling, flowering, grain-filling, and mature periods, 20 Tartary buckwheat plants with consistent growth were randomly excavated in each treatment plot. During the excavation process, the root system was as complete as possible, and the soil loosely combined with the root system was shaken off, collected as the rhizosphere soil sample, and stored in a refrigerator at −20°C for determination of rhizosphere soil enzyme activity and soil nutrient content. The roots of 10 plants were rinsed with running water and cut off after the water was filtered to determine the root morphological index and root activity. The leaves (located on the top 4–6 nodes of the main stem) of the remaining 10 plants were treated with liquid nitrogen for 30 s and then stored in a refrigerator at −80°C.

### 2.3 Measurement

In accordance with Zhang et al. (2023a), Richards’ equation was used to describe the grain-filling process as follows: W=A/(1+Be^-Kt^)1/N, where the W is the grain weight of Tartary buckwheat during grain filling, A is the final grain weight at harvest, B is the coefficient determined by regression analysis, e is a constant, K is the constant growth rate, N is the shape parameter, and t is the time after flowering.


$${\text{G}}_{\text{max}}=\left(\text{KW}/\text{N}\right)\left[1-{\left(\text{W}/\text{A}\right)}^{\text{N}}\right],$$

$${\text{G}}_{\text{mean}}=\text{AK}/\left[2\left(\text{N}+2\right)\right],$$

Where G_max_ is the maximum grain-filling rate and G_mean_ is the mean grain-filling rate.

Crude enzyme solution was extracted according to the following process: 1 g of Tartary buckwheat grains were collected, shelled, weighed, added to 2 mL extract, ground, homogenized, and centrifuged (10000 r min^-1^, 30 min) for testing. The extraction liquid contained 100 mmol/L Tricine–NaOH (pH 7.5), 8 mmol L^-1^ MgCl_2_, 2 mmol L^-1^ EDTA, 12.5% glycerol, 1% PVP-40, and 50 mmol L^-1^ β-mercaptoethanol.The activities of ADP-glucose pyrophosphorylase (AGPase) and soluble starch synthase (SSS) were determined using the method of Yang et al. (2003)Superoxide dismutase (SOD) activity was determined by nitrogen blue tetrazolemethod. In brief, 0.5g of the sample was accurately weighted to prepare SOD crude extract. Phosphoric acid buffer solution, Met solution, NBT solution, EDTA-Na_2_ solution, riboflavin, SOD enzyme crude solution, and distilled water were successively added into a 5mL test tube in a certain proportion and placed under light for 20 min. SOD activity was the calculated by colorimetry at 560 nm (Li, 2000; Zhou et al., 2023a). Peroxidase (POD) activity was determined by guaiacol method. In brief, 0.5g of the sample was weighed and extracted with phosphoric acid buffer. Phosphoric acid buffer, H_2_O_2_, and guaiacol were added and colorimetrized at 470 nm. The enzyme activity unit was 0.01 change of A470 per minute (Li, 2000; Zhou et al., 2023a). Malondialdehyde (MDA) content was measured by thiobarbituric acid method. In brief, 0.2g of Tartary buckwheat leaves were weighed, ground evenly with 2mL of 10% trichloroacetic acid, and centrifuged at 12000 r·min^−1^ for 20 min at 4°C. Afterward, 1mL of the test solution and 1 mL of reaction solution were added. The mixture was shaken, mixed, and kept at 95°C for 15 min. The optical density (OD) value at 450, 532, and 600 nm was measured after cooling (Li, 2000; Zhou et al., 2023a).All roots were imaged and scanned using a roots canner (GXY-A, Zhejiang Tuopu Instrument Co., Ltd). The root length, surface area, and volume were obtained using a root analysis system WinRHIZO (version4.0b, Regent Instruments, Inc.) (Zhang et al., 2023b). The root activity was determined via the 2, 3, 5-triphenyltetrazolium chloride method (Li, 2000).

The activity of urease was determined by the method of Guan (1986). The activity of alkaline phosphatase was determined using a kit, and the kit was purchased from Beijing Solarbio Technology Co., LTD, China (https://www.solarbio.com). The available nitrogen, phosphorus, potassium and the organic matter content were determined using a multichannel intelligent soil nutrient meter (OK-V24, China).

In accordance with the method of Zhou et al. (2023b), the plant height, main stem branch number, main stem node number, grain number per plant, grain weight per plant, and 100-grain weight of Tartary buckwheat were measured. One square meter at the center of each plot (not sampled during the experiment, excluding border plants) was randomly selected, and the grains of all Tartary buckwheat plants were collected to determine the yield after air drying (Zhou et al., 2023a).

### 2.4 Statistical analysis

Data were processed using Microsoft Excel 2020 and SPSS20.0. One-way ANOVA was performed, and means were compared using the least significant difference at the 0.05 probability level. The results of 2021 and 2022 were similar. Therefore, the data were presented as the average across the two study years, and the data of both years were deposited as **Supplementary Data**.

## 3 Results

### 3.1 Effects of different potassium fertilizer application rates on the agronomic traits of thin-shelled Tartary buckwheat

**Figure 1:**
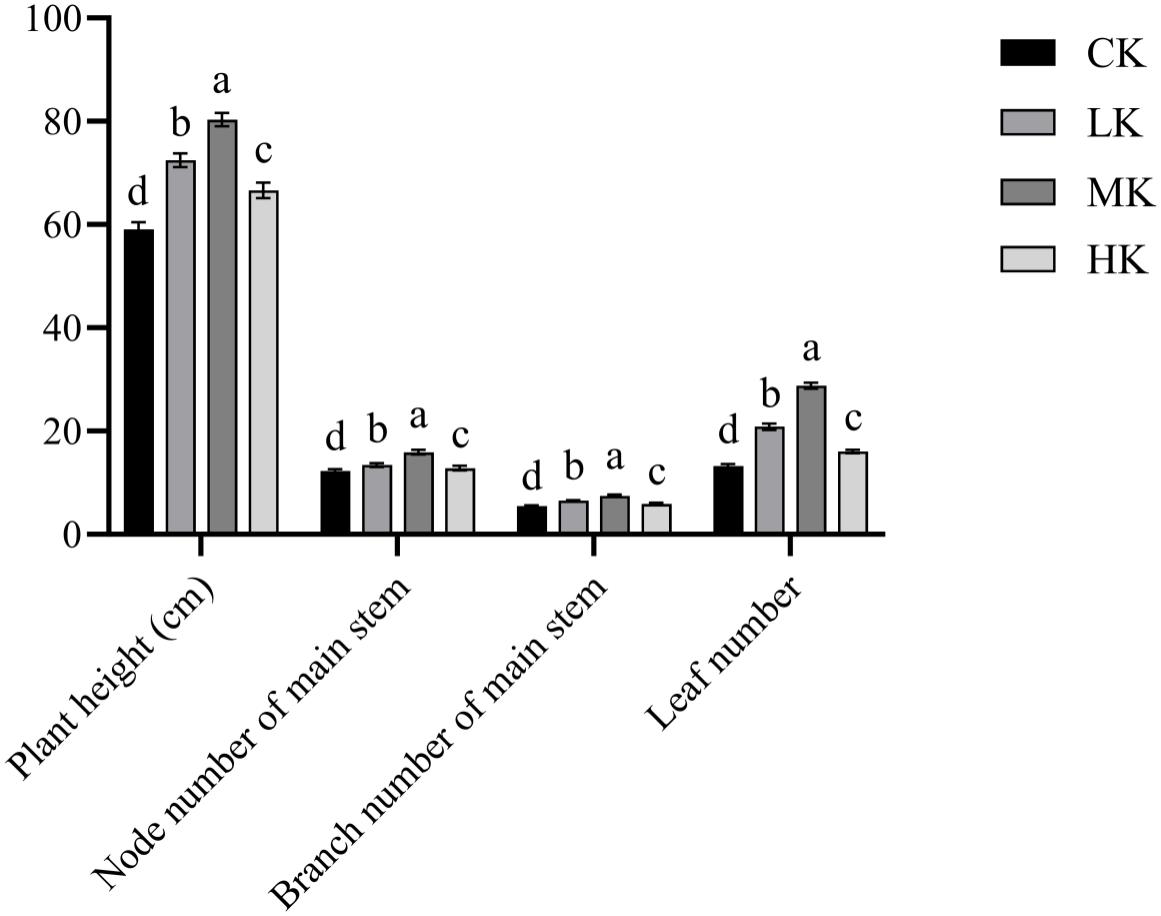
Effects of different potassium fertilizer application rates on agronomic traits of thin-shelled Tartary buckwheat. Small letter in the same column means significant difference at *p*< 0.05. CK: potassium fertilizer application rate was 0 kg·ha^-1^; LK: potassium fertilizer application rate was 15 kg·ha^-1^; MK: potassium fertilizer application rate was 30 kg·ha^-1^; HK: potassium fertilizer application rate was 45 kg·ha^-1^.

The plant height, node number of main stem, branch number of main stem, and leaf number of thin-shelled Tartary buckwheat increased first and then decreased with the increase in potassium fertilizer application rate, and they were significantly higher in MK treatment (**Figure 1**). Compared with CK treatment, MK treatment increased the plant height, main stem node number, main stem branch number, and leaf number by 36.0%, 29.7%, 36.7%, and 118.0%, respectively.

### 3.2 Effects of different potassium fertilizer application rates on the yield of thin-shelled Tartary buckwheat

**Figure 2:**
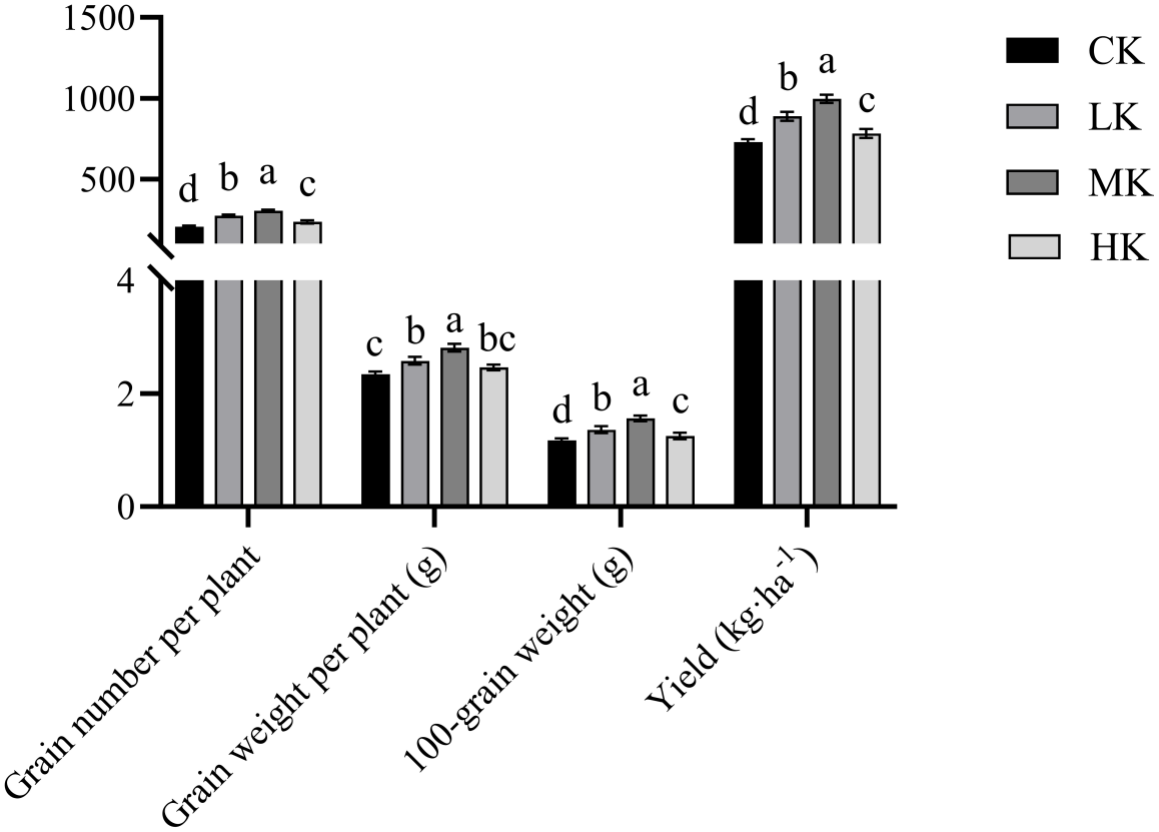
Effects of different potassium fertilizer application rates on yield of thin-shelled Tartary buckwheat. Small letter in the same column means significant difference at *p*< 0.05. CK: potassium fertilizer application rate was 0 kg·ha^-1^; LK: potassium fertilizer application rate was 15 kg·ha^-1^; MK: potassium fertilizer application rate was 30 kg·ha^-1^; HK: potassium fertilizer application rate was 45 kg ha^-1^.

The number of grains per plant, grain weight per plant, 100-grain weight, and yield of thin-shelled Tartary buckwheat increased first and then decreased with the increase in potassium fertilizer application, and they were significantly higher in MK treatment (**Figure 2**). Compared with CK treatment, MK treatment increased the grain number per plant, grain weight per plant, 100-grain weight, and yield by 49.1%, 20.1%, 33.3%, and 36.7%, respectively.

### 3.3 Effects of different potassium fertilizer application rates on the gain weight and grain-filling rate of thin-shelled Tartary buckwheat

**Figure 3:**
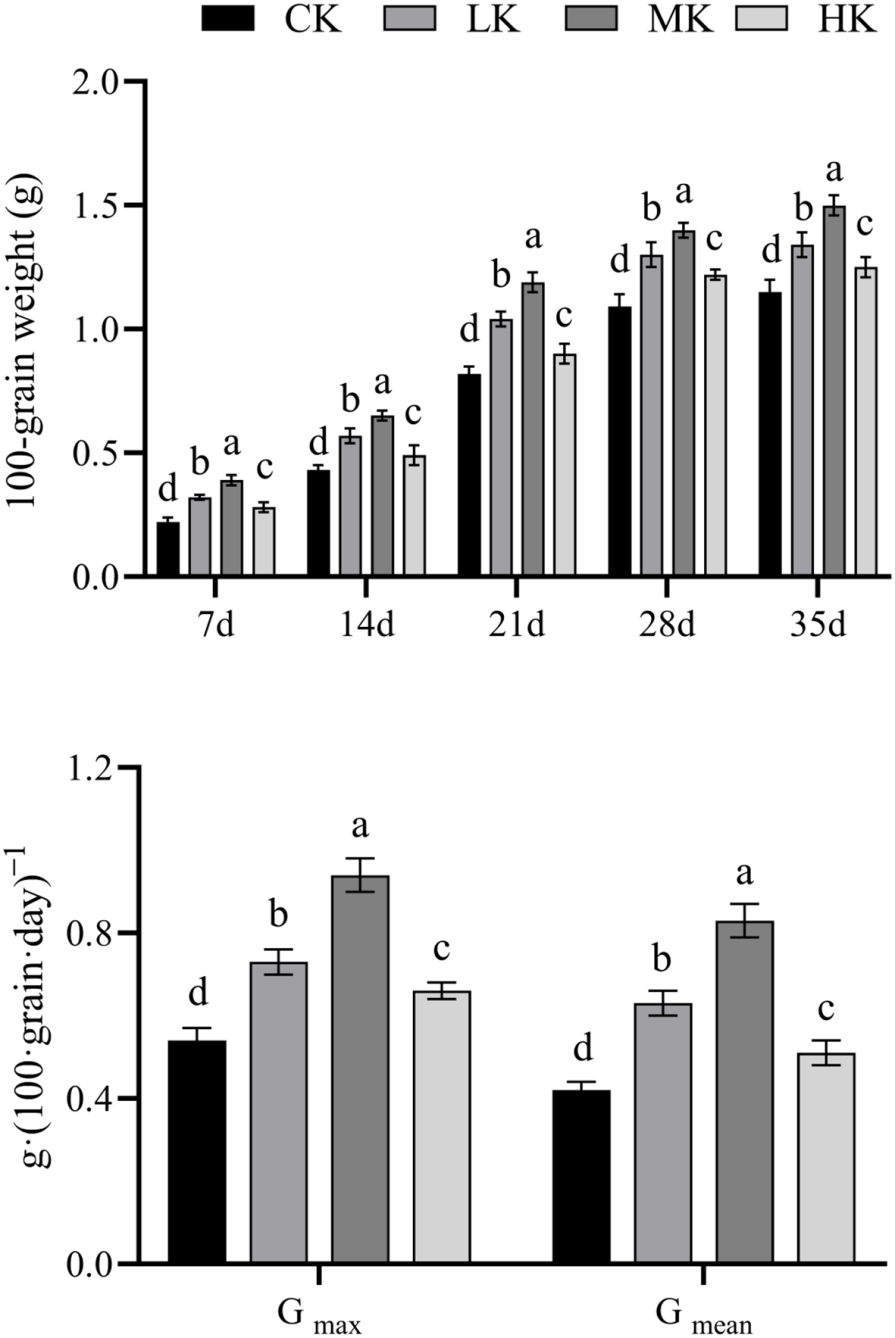
Effects of different potassium fertilizer application rates on gain weight (g) and grain-filling rate of thin-shelled Tartary buckwheat. Small letter in the same column means significant difference at p< 0.05. CK: potassium fertilizer application rate was 0 kg·ha^-1^; LK: potassium fertilizer application rate was 15 kg·ha^-1^; MK: potassium fertilizer application rate was 30 kg·ha^-1^; HK: potassium fertilizer application rate was 45 kg·ha^-1^. G_max:_ the maximum grain-filling rate; G_mean_: the mean grain-filling rate.

The 100-grain weight increased continuously with the advancement of growth period (**Figure 3**). The 100-grain weight of MK treatment was significantly higher than those of the other three treatments. The *G_max_* and *G_mean_* were significantly higher in MK treatment than in other treatments and the lowest in CK treatment. Compared with CK treatment, MK treatment increased the *G_max_* and *G_mean_* by 74.1% and 97.6%, respectively.

### 3.4 Effects of different potassium fertilizer application rates on starch synthase in thin-shelled Tartary buckwheat grains

**Figure 4:**
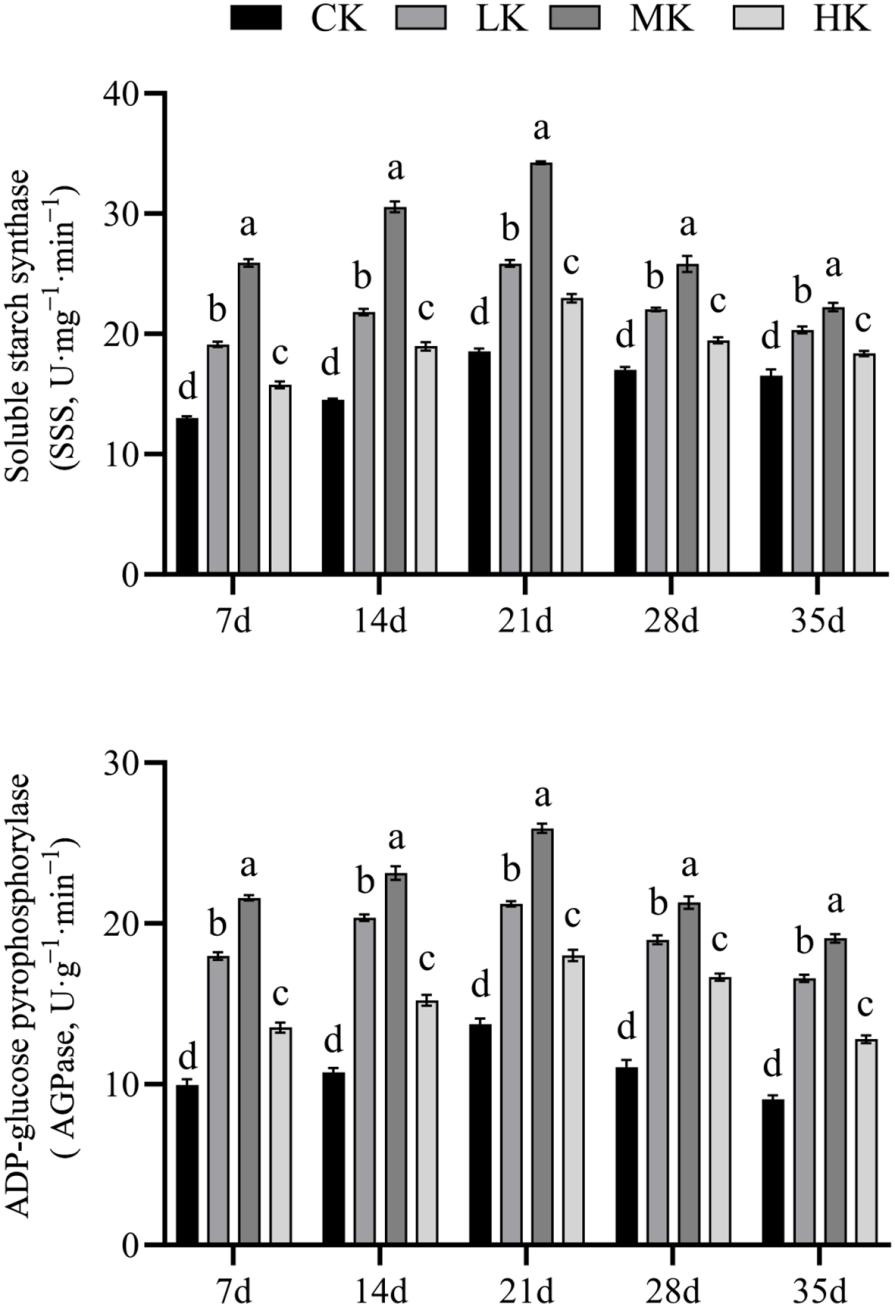
Effects of different potassium fertilizer application rates on starch synthase in grains of thin-shelled Tartary buckwheat. Small letter in the same column means significant difference at *p*< 0.05. CK: potassium fertilizer application rate was 0 kg·ha^-1^; LK: potassium fertilizer application rate was 15 kg·ha^-1^; MK: potassium fertilizer application rate was 30 kg·ha^-1^; HK: potassium fertilizer application rate was 45 kg·ha^-1^.

The activities of AGPase and SSS in grains increased first and then decreased with the advancement of growth period (**Figure 4**). With the increase in potassium fertilizer application rate, the activities of AGPase and SSS in grains increased first and then decreased. They were significantly higher in MK treatment than in other treatments. Compared with CK treatment, MK treatment increased the activities of SSS and AGPase by average of 20.0% and 39.7%, respectively.

### 3.5 Effects of different potassium fertilizer application rates on the antioxidant enzyme activity and MDA content in leaves of thin-shelled Tartary buckwheat

**Figure 5:**
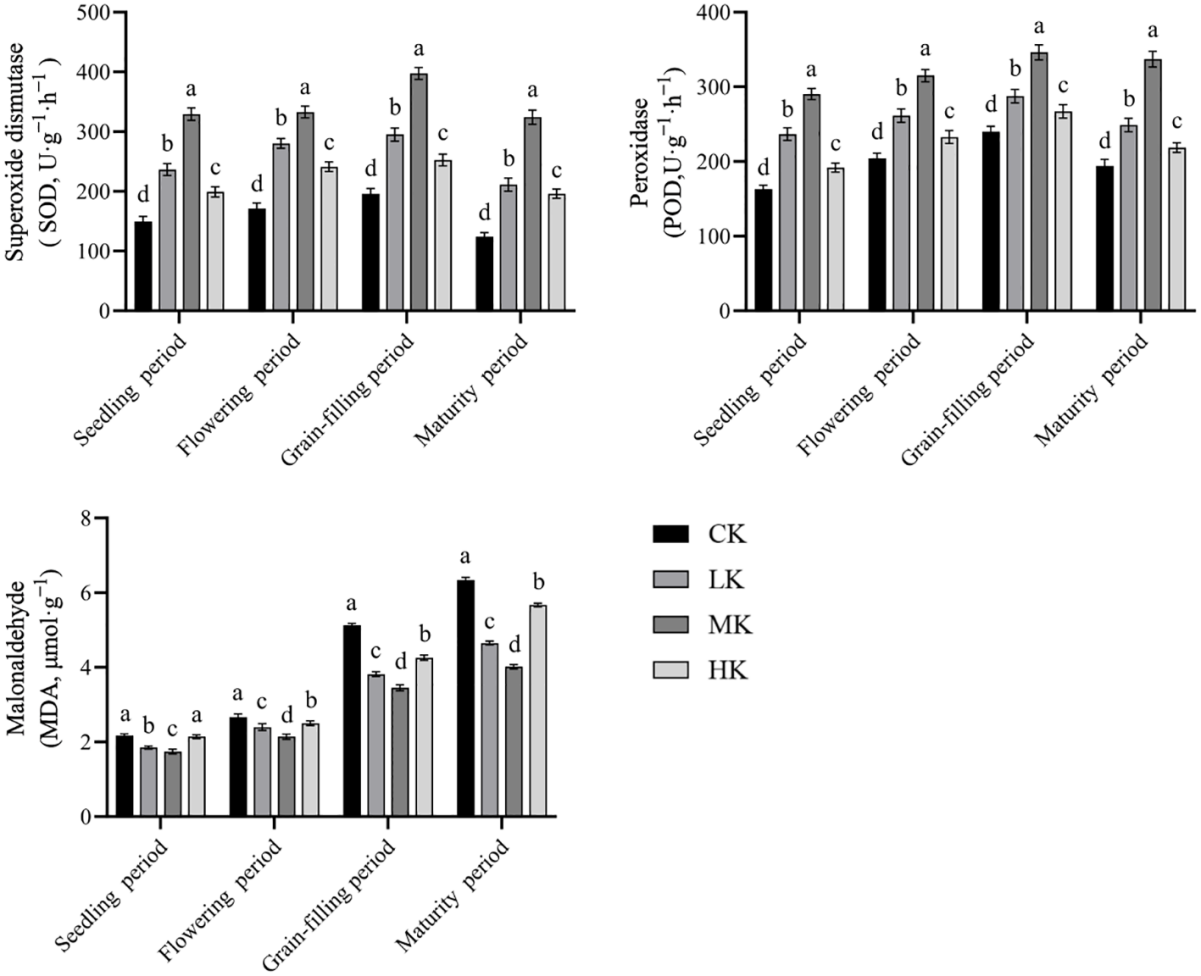
Effects of different potassium fertilizer application rates on antioxidant enzyme activity and MDA content of thin-shelled Tartary buckwheat. Small letter in the same column means significant difference at *p*< 0.05. CK: potassium fertilizer application rate was 0 kg·ha^-1^; LK: potassium fertilizer application rate was 15 kg·ha^-1^; MK: potassium fertilizer application rate was 30 kg ha^-1^; HK: potassium fertilizer application rate was 45 kg·ha^-1^.

The activities of SOD and POD in leaves increased first and then decreased with the advancement of growth period and reached the maximum at grain-filling period (**Figure 5**). The content of MDA showed a trend of continuous increase. With the increase in potassium fertilizer application rate, the activities of SOD and POD increased first and then decreased, and those activities in MK treatment were significantly higher than in the other three treatments. The MDA content decreased first and then increased with the increase in potassium fertilizer application rate. Compared with CK treatment, MK treatment increased the activities of SOD and POD by average of 116.0% and 60.9%, whereas decreased the content of MDA by average of 30.4%.

### 3.6 Effects of different potassium fertilizer application rates on the root morphology and root activity of thin-shelled Tartary buckwheat

**Figure 6:**
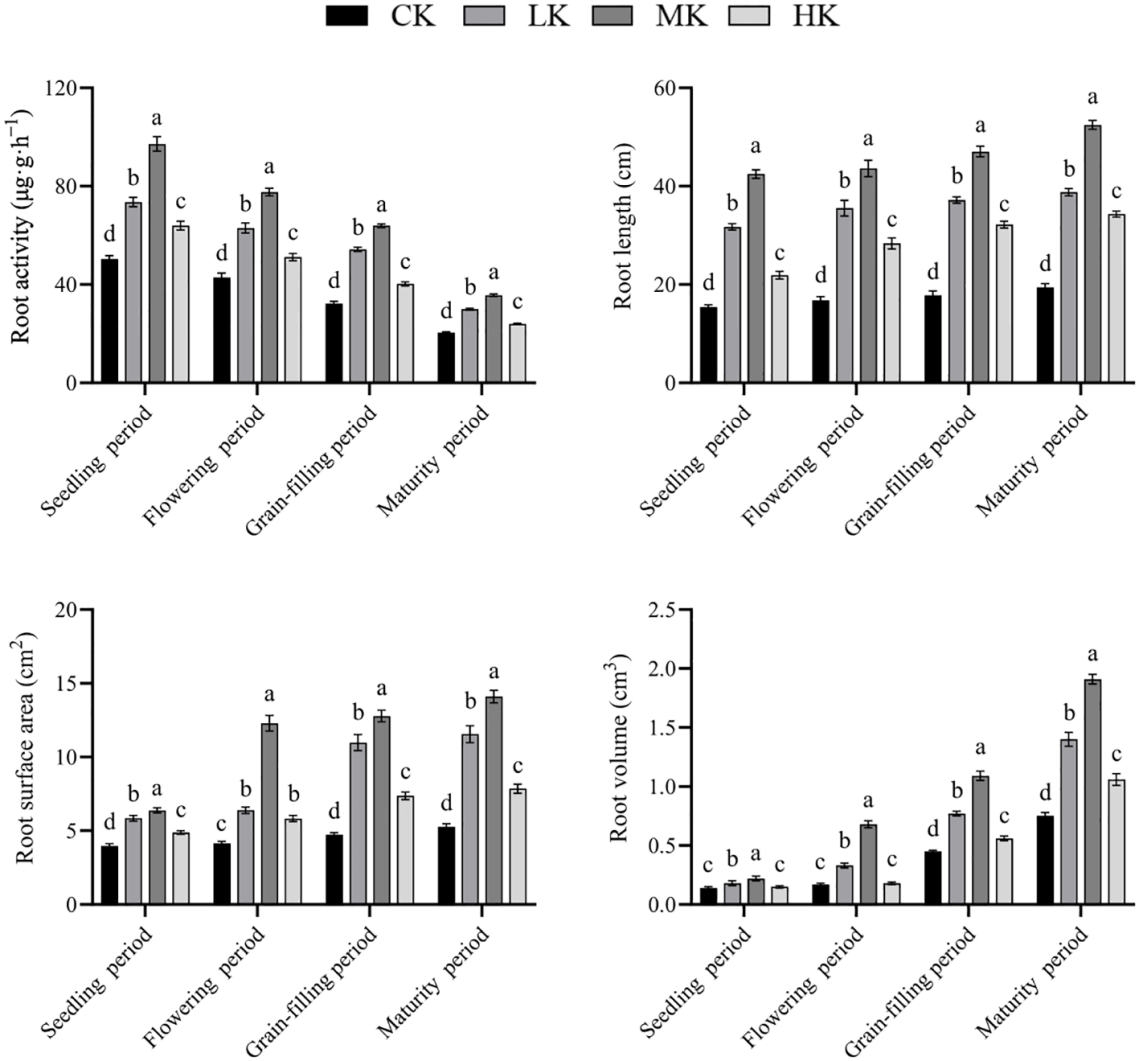
Effects of different potassium fertilizer application rates on root morphology and root activity of thin-shelled Tartary buckwheat. Small letter in the same column means significant difference at *p*< 0.05. CK: potassium fertilizer application rate was 0 kg·ha^-1^; LK: potassium fertilizer application rate was 15 kg·ha^-1^; MK: potassium fertilizer application rate was 30 kg·ha^-1^; HK: potassium fertilizer application rate was 45 kg·ha^-1^.

The root length, root surface area, and root volume of thin-shelled Tartary buckwheat increased continuously with the advancement of growth period (**Figure 6**). Meanwhile, the root activity decreased continuously with the advancement of growth period. With the increase in potassium fertilizer application rate, the root activity, root length, root surface area, and root volume increased first and then decreased. Compared with CK treatment, MK treatment increased the root activity, root length, root surface area, and root volume by average of 87.9%, 167.1%, 151.1%, and 158.3%, respectively.

### 3.7 Effects of different potassium fertilizer application rates on the available nutrients and soil enzyme activities in rhizosphere soil of thin-shelled Tartary buckwheat

**Figure 7:**
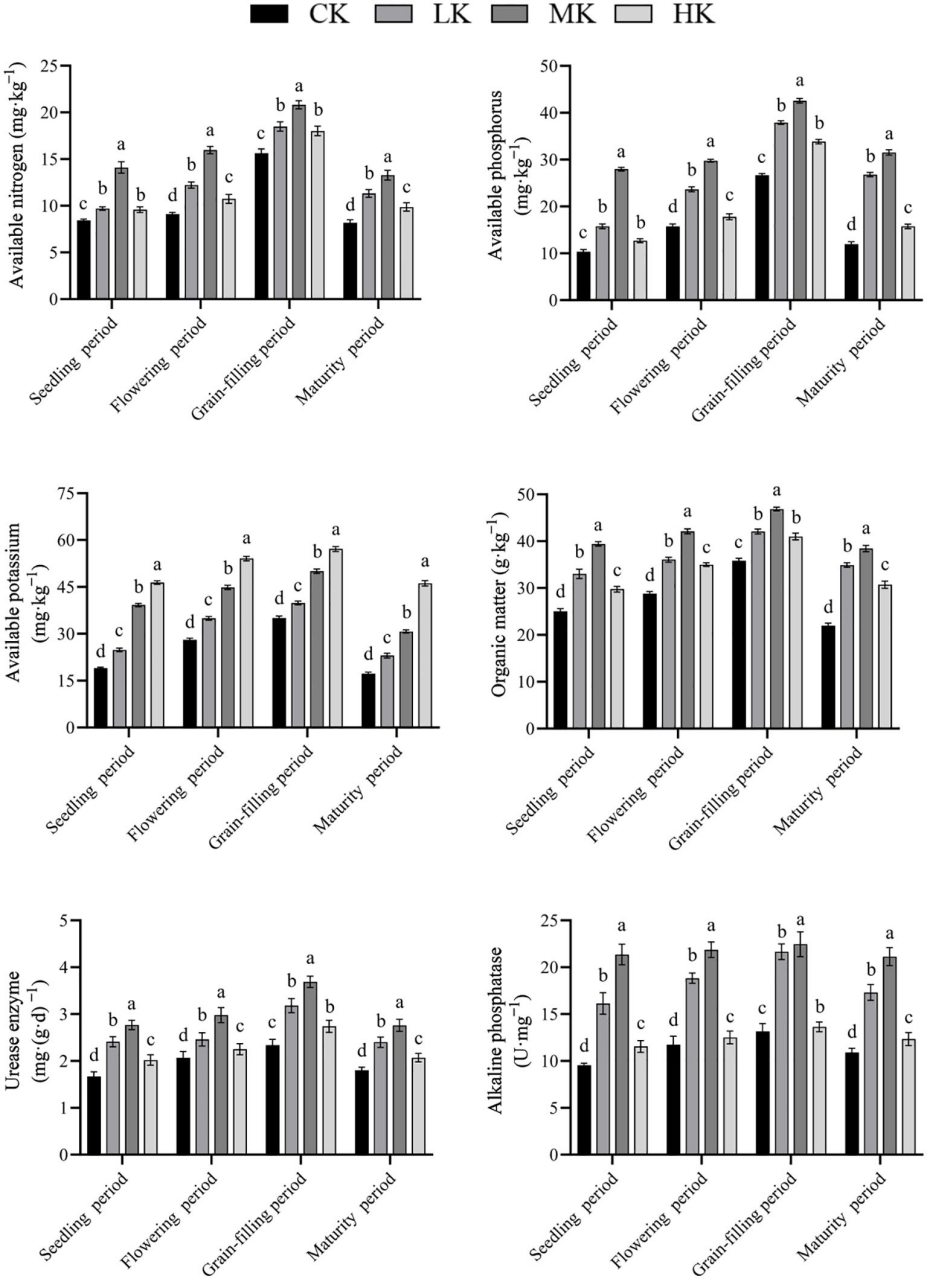
Effects of different potassium fertilizer application rates on available nutrients and soil enzyme activities in rhizosphere soil. Small letter in the same column means significant difference at *p*< 0.05. CK: potassium fertilizer application rate was 0 kg·ha^-1^; LK: potassium fertilizer application rate was 15 kg·ha^-1^; MK: potassium fertilizer application rate was 30 kg·ha^-1^; HK: potassium fertilizer application rate was 45 kg·ha^-1^.

The contents of available nitrogen, phosphorus, and potassium and organic matter content and the activities of urease and alkaline phosphatase increased first and then decreased with the advancement of growth period (**Figure 7**). With the increase in potassium fertilizer application rate, the contents of available nitrogen, available phosphorus, and organic matter and the activities of urease and alkaline phosphatase increased first and then decreased. However, the content of available potassium increased continuously with the increase in potassium fertilizer application rate. Compared with CK treatment, MK treatment increased the contents of available nitrogen, available phosphorus, and organic matter and the activities of urease and alkaline phosphatase by average of 55.2%, 103.5%, 49.5%, 54.8%, and 91.4%, respectively. Compared with CK treatment, HK treatment increased the contents of available potassium by average of 105.4%.

## 4 Discussion

The grain filling process is closely related to the grain weight and final yield of crops. The grain weight of crops mainly depends on the grain filling rate, and a higher average grain filling rate is a prerequisite for obtaining a larger grain weight (Zhang et al., 2023a). Zhang et al. (2023a) found that compared with CK treatment, apical dominance removal treatment can significantly increase the G_max_ and G_mean_ of common buckwheat, thus promoting the grain filling and increasing the grain weight. Guo et al. (2022) found that appropriate straw returning combined with chemical fertilizer can increase the G_max_ and G_mean_ of common buckwheat grains, thus promoting the grain filling and increasing the grain weight and final yield. Liu et al. (2024) found that suitable potassium fertilizer application can promote the grain filling of *Coixlacryma-jobi* L. and increase the grain weight. In the present study, compared with CK treatment, potassium fertilizer treatment significantly increased the G_max_ and G_mean_ of thin-shelled Tartary buckwheat and significantly increased the grain weight, especially MK treatment. This finding is consistent with the abovementioned research results. These phenomena likely occurred because suitable potassium fertilizer application can promote the absorption of rhizosphere soil nutrients by the roots of thin-shelled Tartary buckwheat for enough nutrients to supply the growth during grain-filling period, thus improving the G_max_ and G_mean_ of grains, increasing the accumulation of grain filling substances, and promoting the grain filling; therefore, the grains obtain more photosynthetic products, thereby increasing grain weight (Zheng et al., 2017). Zhang et al. (2020) found that an appropriate nitrogen fertilizer treatment can increase the activities of AGPase and SSS in Tartary buckwheat grains and promote grain filling. The results of the present study showed that compared with CK treatment, potassium fertilizer treatment significantly increased the activities of AGPase and SSS in the grains of thin-shelled Tartary buckwheat, and the activity was the strongest under MK treatment, which is consistent with the above research results. The reason may be that the maximum sink strength of grains that reached under MK treatment, the more photosynthetic substances accumulated in stems and leaves can be transported to grains, which is conducive to increase grain filling and grain weight. The finding showed that the enhancement of the physiological activity of grains during grain-filling period is an important physiological reason for potassium fertilizer treatment, especially MK treatment, to promote grain filling and increase grain weight.

The senescence of crops can cause leaf yellowing, severe shortening of functional period, and cessation of photosynthesis, which all, in turn, seriously affect the growth and final yield formation of crops (Abeledo et al., 2020). The activity of antioxidant enzymes, such as SOD and POD, in leaves affects the speed of senescence. The higher the activity, the stronger the ability of crops to remove their own harmful substances, and the slower the senescence process (Zhou et al., 2023a). MDA content is also closely related to plant senescence (Chen et al., 2018). A study showed that the root activity of crops affected the senescence process of aboveground leaves, and leaf senescence can be delayed by appropriately increasing the root activity of crops (Zhou et al., 2023a). Wang et al. (2016) showed that senescence reduced the activity of antioxidant enzymes, such as SOD, in plant leaves. This phenomenon resulted in the dynamic balance between the production and scavenging of reactive oxygen species in the body being broken, leading to a large accumulation of reactive oxygen species and then membrane damage or destruction, ultimately resulting in an increase in MDA content. Wang et al. (2020) found that continuous cropping led to decreased SOD and POD activities and increased MDA content in common buckwheat leaves, which aggravated the senescence and reduced the yield. Sun and Teng (2017) found that the application of appropriate potassium fertilizer could increase the antioxidant enzyme activity of wheat, delay the anti-senescence ability of plants in the middle and late periods of grain filling, and then increase the yield. Ren et al. (2018) found that the application of appropriate potassium fertilizer could increase the activities of SOD and POD in tobacco and effectively enhance the resistance and anti-senescence ability of tobacco leaves. In the present study, compared with CK treatment, the application of potassium fertilizer increased the activities of SOD and POD in the leaves in the middle position of Tartary buckwheat, increased the root activity, and decreased the content of MDA. Among the treatments, MK treatment was the best, which is consistent with the above research results, indicating that the application of suitable potassium fertilizer could delay the senescence of thin-shelled Tartary buckwheat leaves. However, the senescence was aggravated with increased potassium fertilizer application. The reason may be that the application of high potassium caused a nutritional imbalance among nitrogen, phosphate, and potassium, resulting in accelerated senescence of Tartary buckwheat. Studies have shown that assimilates produced by the leaves in the middle position of Tartary buckwheat have the highest contribution to grain filling (Zhang et al., 2023a). Therefore, the photosynthetic capacity of these leaves determines the yield of Tartary buckwheat. The senescence of these leaves can cause their yellowing and decrease the photosynthetic capacity, resulting in insufficient filling materials in late grain-filling period and poor filling degree (Guo et al., 2022). This phenomenon may be the reason that appropriate potassium fertilizer application can increase the final grain weight and yield.

Potassium is an indispensable nutrient element for crop growth and development. It participates in organic matter synthesis and photosynthesis and assimilate transport. It also plays an important role in yield formation. A study has shown that potassium application can significantly increase the grain yield of rice (Deng et al., 2023). Liu et al. (2023) found that straw returning combined with appropriate potassium fertilizer could significantly increase the yield of maize. In the present study, potassium application significantly increased the yield of thin-shelled Tartary buckwheat, and MK treatment had the best effect, which was 1.37 times that of CK treatment, consistent with the results of Wang et al. (2019). It may be that appropriate potassium fertilizer application is beneficial to the absorption of rhizosphere soil’s available nutrients by the roots of thin-shelled Tartary buckwheat. It also promotes root growth (**Figure 6**); increases the plant height, main stem node number, main stem branch number, and leaf number of thin-shelled Tartary buckwheat; and promotes the accumulation and transfer of dry matter, thereby increasing the aboveground biomass and yield. Notably, with the further increase in potassium fertilizer application rate, the yield of thin-shelled Tartary buckwheat decreased, may be because when excessive potassium fertilizer was applied, the absorption of nitrogen, phosphorus, and potassium in rhizosphere soil was inhibited. Furthermore, the absorption of magnesium in rhizosphere soil by thin-shelled Tartary buckwheat was inhibited, so the population photosynthesis of crops decreased, resulting in a decrease in final yield (Wang et al., 2019).

## 5 Conclusions

Appropriate potassium fertilizer application (30kg·ha^−1^) can increase the maximum and average grain-filling rates and increase the activities of AGPase and SSS in grains, thus promoting the grain filling and increasing the grain weight. Appropriate potassium fertilizer application (30kg·ha^−1^) can improve the SOD and POD activities of thin-shelled Tartary buckwheat leaves and reduce MDA content, thus delaying senescence. It can also promote the growth of root and the absorption of rhizosphere soil nutrients, increase the plant height and leaf number; promote the accumulation and transfer of dry matter, thus finally increasing the yield of thin-shelled Tartary buckwheat.

## Data availability statement

The original contributions presented in the study are included in the article/**Supplementary Material**. Further inquiries can be directed to the corresponding author.

## Author contributions

LT: Investigation, Methodology, Writing – original draft, Writing – review & editing. JT: Investigation, Methodology, Writing – original draft, Writing – review & editing. KH: Conceptualization, Funding acquisition, Investigation, Writing – original draft, Writing – review & editing. XH: Funding acquisition, Writing – review & editing.
