# Supplementary material for: Potassium fertilizer promotes the thin-shelled Tartary buckwheat yield by delaying senescence and promoting grain filling
Source: Front Plant Sci. 2024 May 2;15:1385548. doi: 10.3389/fpls.2024.1385548 (PMC11096533; doi:10.3389/fpls.2024.1385548)
Supplement: Supplementary file 1 [file Table_1.docx]

Supplementary Material

**Supplementary data in 2020**

**TABLE S1 Effects of different potassium fertilizer application rates on agronomic traits of thin-shelled Tartary buckwheat**

| Treatment | Plant  height (cm) | Node number  of main stem | Branch number of  main stem | Leaf number |
| --- | --- | --- | --- | --- |
| CK | 58.73±1.50d | 12.21±0.25c | 5.32±0.11c | 12.42±0.39d |
| LK | 71.57±1.36b | 12.73±0.39b | 5.77±0.16b | 20.61±0.67b |
| MK | 79.13±1.27a | 13.92±0.36a | 6.28±0.23a | 23.24±0.59a |
| HK | 65.57±1.12c | 12.68±0.34b | 5.45±0.14c | 15.63±0.32c |

Data are presented as mean ± standard error of the mean. Small letter in the same column means significant difference at *p* < 0.05. CK: potassium fertilizer application rate was 0 kg·ha^-1^; LK: potassium fertilizer application rate was 15 kg·ha^-1^; MK: potassium fertilizer application rate was 30 kg·ha^-1^; HK: potassium fertilizer application rate was 45 kg·ha^-1^

**TABLE S2** **Effects of different potassium fertilizer application rates on yield of thin-shelled Tartary buckwheat**

| Treatment | Grain number  per plant | Grain weight  per plant (g) | 100-grain weight  (g) | Yield  (kg·ha^-1^) |
| --- | --- | --- | --- | --- |
| CK | 201.6±8.4d | 2.26±0.05c | 1.15±0.04d | 714.2±14.9d |
| LK | 265.2±8.3b | 2.48±0.06b | 1.34±0.07b | 882.6±29.7b |
| MK | 303.4±9.1a | 2.77±0.05a | 1.49±0.05a | 967.4±26.2a |
| HK | 220.1±9.8c | 2.40±0.04b | 1.24±0.07c | 752.3±28.8c |

Data are presented as mean ± standard error of the mean. Small letter in the same column means significant difference at *p* < 0.05. CK: potassium fertilizer application rate was 0 kg·ha^-1^; LK: potassium fertilizer application rate was 15 kg·ha^-1^; MK: potassium fertilizer application rate was 30 kg·ha^-1^; HK: potassium fertilizer application rate was 45 kg·ha^-1^.

**TABLE S3 Effects of different potassium fertilizer application rates on gain weight (g) and grain-filling rate of thin-shelled Tartary buckwheat**

| Treatment | [Period](javascript:;) | | | | | G _max._  g (100·grain· day)^−1^ | G _mean_  g (100·grain·day)^−1^ |
| --- | --- | --- | --- | --- | --- | --- | --- |
|  | 7d | 14d | 21d | 28d | 35d |  |  |
| CK | 0.21±0.02d | 0.42±0.02d | 0.81±0.02d | 1.08±0.04d | 1.10±0.04d | 0.53±0.04d | 0.41±0.03d |
| LK | 0.32±0.02b | 0.56±0.04b | 1.02±0.03b | 1.29±0.04b | 1.30±0.04b | 0.64±0.03b | 0.54±0.03b |
| MK | 0.38±0.01a | 0.65±0.03a | 1.13±0.04a | 1.38±0.02a | 1.39±0.03a | 0.92±0.05a | 0.82±0.04a |
| HK | 0.27±0.02c | 0.47±0.04c | 0.90±0.04c | 1.20±0.02c | 1.23±0.03c | 0.60±0.02c | 0.50±0.03c |

Data are presented as mean ± standard error of the mean. Small letter in the same column means significant difference at p < 0.05. CK: potassium fertilizer application rate was 0 kg·ha^-1^; LK: potassium fertilizer application rate was 15 kg·ha^-1^; MK: potassium fertilizer application rate was 30 kg·ha^-1^; HK: potassium fertilizer application rate was 45 kg·ha^-1^. G_max:_ the maximum grain-filling rate; G_mean_: the mean grain-filling rate.

**TABLE S4** **Effects of different potassium fertilizer application rates on starch synthase in grains of thin-shelled Tartary buckwheat**

| Index | Treatment | Period | | | | |
| --- | --- | --- | --- | --- | --- | --- |
|  |  | 7d | 14d | 21d | 28d | 35d |
| Soluble starch synthase  (SSS, U·mg^-1^·min^−1^) | CK | 12.92±0.12d | 14.22±0.11d | 18.34±0.23d | 16.88±0.23d | 16.22±0.46d |
|  | LK | 19.02±0.23b | 20.71±0.29b | 25.29±0.33b | 21.73±0.11b | 19.96±0.19b |
|  | MK | 25.65±0.33a | 29.55±0.45a | 33.21±0.12a | 24.81±0.56a | 22.05±0.48a |
|  | HK | 14.02±0.29c | 18.63±0.41c | 22.26±0.31c | 19.30±0.28c | 18.11±0.21c |
| ADP-glucose pyrophosphorylase  (AGPase, U·g^-1^·min^−1^) | CK | 9.89±0.31d | 10.02±0.21d | 13.21±0.24d | 10.91±0.34d | 8.98±0.29d |
|  | LK | 17.73±0.23b | 19.31±0.13b | 19.76±0.14b | 18.86±0.28b | 16.23±0.19b |
|  | MK | 20.58±0.19a | 21.62±0.47a | 25.52±0.23a | 20.24±0.39a | 18.63±0.26a |
|  | HK | 12.88±0.39c | 14.57±0.23c | 17.98±0.25c | 16.34±0.12c | 12.49±0.22c |

Data are presented as mean ± standard error of the mean. Small letter in the same column means significant difference at *p* < 0.05. CK: potassium fertilizer application rate was 0 kg·ha^-1^; LK: potassium fertilizer application rate was 15 kg·ha^-1^; MK: potassium fertilizer application rate was 30 kg·ha^-1^; HK: potassium fertilizer application rate was 45 kg·ha^-1^.

**TABLE S5** **Effects of different potassium fertilizer application rates on antioxidant enzyme activity and MDA content of thin-shelled Tartary buckwheat**

| Index | Treatment | Period | | | |
| --- | --- | --- | --- | --- | --- |
|  |  | Seedling period | Flowering period | Grain-filling period | Maturity period |
| Superoxide dismutase  ( SOD, U·g^-1^·h^-1^ ) | CK | 143.23±9.34d | 163.01±9.34d | 188.57±9.23d | 114.79±5.98d |
|  | LK | 232.63±10.98b | 279.08±8.22b | 290.54±8.66b | 204.45±7.98b |
|  | MK | 309.33±12.21a | 313.44±10.13a | 391.53±11.21a | 317.76±11.03a |
|  | HK | 196.69±7.36c | 240.63±9.98c | 244.96±9.09c | 185.78±7.65c |
| Peroxidase  (POD,U·g^-1^·h^-1^) | CK | 152.28±6.34d | 197.15±5.76d | 236.56±7.98d | 192.78±7.87d |
|  | LK | 228.31±7.11b | 251.91±7.98b | 283.99±9.39b | 245.7±9.09b |
|  | MK | 279.23±9.32a | 308.10±8.12a | 342.04±11.98a | 331.17±10.87a |
|  | HK | 188.19±5.98c | 223.19±9.65c | 259.33±8.34c | 214.06±6.05c |
| Malonaldehyde (MDA, μmol·g^−1^) | CK | 2.45±0.04a | 3.26±0.08a | 6.30±0.06a | 7.50±0.07a |
|  | LK | 1.94±0.03b | 2.88±0.09c | 4.81±0.05c | 5.07±0.06c |
|  | MK | 1.86±0.07c | 2.51±0.08d | 4.16±0.09d | 4.74±0.06d |
|  | HK | 2.42±0.06a | 3.02±0.07b | 5.32±0.08b | 6.61±0.03b |

Data are presented as mean ± standard error of the mean. Small letter in the same column means significant difference at *p* < 0.05. CK: potassium fertilizer application rate was 0 kg·ha^-1^; LK: potassium fertilizer application rate was 15 kg·ha^-1^; MK: potassium fertilizer application rate was 30 kg·ha^-1^; HK: potassium fertilizer application rate was 45 kg·ha^-1^.

**TABLE S6 Effects of different potassium fertilizer application rates on root morphology and root activity of thin-shelled Tartary buckwheat**

| Index | Treatment | Period | | | |
| --- | --- | --- | --- | --- | --- |
|  |  | Seedling period | Flowering period | Grain-filling period | Maturity period |
| Root activity  (μg·g·h^-1^) | CK | 48.72±1.28d | 41.98±1.03d | 31.40±0.92d | 19.65±0.14d |
|  | LK | 71.32±1.16b | 62.62±1.46b | 52.36±0.85b | 27.24±0.12b |
|  | MK | 96.04±2.01a | 76.91±1.26a | 63.47±1.03a | 34.87±0.16a |
|  | HK | 59.25±1.25c | 50.28±1.18c | 38.91±0.61c | 22.51±0.11c |
| Root length  (cm) | CK | 14.40±0.48d | 15.40±0.32d | 16.24±0.65d | 17.81±0.65d |
|  | LK | 30.54±0.54b | 34.09±0.94b | 35.70±0.56b | 37.08±0.97b |
|  | MK | 40.08±1.21a | 42.62±1.08a | 45.89±1.21a | 49.65±0.98a |
|  | HK | 21.23±0.74c | 24.79±0.59c | 31.02±0.54c | 32.55±0.44c |
| Root surface area  (cm^2^ ) | CK | 3.88±0.18d | 3.97±0.14d | 4.20±0.12d | 4.85±0.14d |
|  | LK | 5.74±0.28b | 6.30±0.28b | 10.80±0.49b | 11.06±0.45b |
|  | MK | 6.33±0.24a | 12.15±0.52a | 12.48±0.46a | 13.96±0.57a |
|  | HK | 4.74±0.11c | 5.69±0.13c | 7.09±0.38c | 7.78±0.28c |
| Root volume  (cm^3^) | CK | 0.13±0.01c | 0.15±0.01c | 0.44±0.02d | 0.73±0.04d |
|  | LK | 0.16±0.02b | 0.32±0.01b | 0.71±0.01b | 1.37±0.07b |
|  | MK | 0.21±0.01a | 0.64±0.02a | 1.06±0.04a | 1.88±0.02a |
|  | HK | 0.14±0.01c | 0.17±0.01c | 0.53±0.02c | 1.02±0.05c |

Data are presented as mean ± standard error of the mean. Small letter in the same column means significant difference at *p* < 0.05. CK: potassium fertilizer application rate was 0 kg·ha^-1^; LK: potassium fertilizer application rate was 15 kg·ha^-1^; MK: potassium fertilizer application rate was 30 kg·ha^-1^; HK: potassium fertilizer application rate was 45 kg·ha^-1^.

**TABLE S7 Effects of different potassium fertilizer application rates on available nutrients and soil enzyme activities in rhizosphere soil**

| Index | Treatment | Period | | | |
| --- | --- | --- | --- | --- | --- |
|  |  | Seedling period | Flowering period | Grain-filling period | Maturity period |
| Available nitrogen  (mg·kg^-1^) | CK | 8.23±0.18c | 8.98±0.25d | 15.46±0.36d | 8.13±0.28d |
|  | LK | 9.45±0.24b | 11.02±0.28b | 18.18±0.42b | 11.26±0.29b |
|  | MK | 13.96±0.56a | 15.37±0.36a | 20.57±0.38a | 12.87±0.34a |
|  | HK | 9.27±0.32b | 10.14±0.32c | 17.93±0.48c | 9.79±0.21c |
| Available phosphorus  (mg·kg^-1^) | CK | 10.26±0.65d | 15.47±0.39d | 21.35±0.26d | 11.88±0.34d |
|  | LK | 15.57±0.39b | 23.23±0.28b | 36.71±0.34b | 26.67±0.21b |
|  | MK | 27.58±0.18a | 29.37±0.12a | 38.81±0.28a | 30.65±0.42a |
|  | HK | 12.55±0.29c | 17.61±0.48c | 32.78±0.25c | 15.58±0.22c |
| Available potassium  (mg·kg^-1^) | CK | 18.81±0.21d | 23.15±0.38d | 31.27±0.38d | 16.51±0.42d |
|  | LK | 24.71±0.17c | 34.67±0.58c | 38.42±0.24c | 22.72±0.68c |
|  | MK | 38.25±0.25b | 44.28±0.62b | 49.43±0.52b | 29.62±0.21b |
|  | HK | 45.14±0.48a | 51.19±0.45a | 54.63±0.75a | 45.55±0.93a |
| Organic matter  (g·kg^-1^) | CK | 24.21±0.58d | 28.31±0.25c | 35.46±0.25c | 21.81±0.32d |
|  | LK | 32.99±0.99b | 35.28±0.41b | 41.61±0.21b | 34.80±0.54b |
|  | MK | 38.25±0.13a | 41.29±0.58a | 46.38±0.28a | 37.92±0.57a |
|  | HK | 29.44±0.54c | 34.86±0.36b | 40.95±0.49b | 30.27±0.69c |
| Urease enzyme  mg (g·d)^-1^ | CK | 1.65±0.13d | 2.05±0.13c | 2.23±0.11d | 1.76±0.08d |
|  | LK | 2.39±0.15b | 2.37±0.16b | 3.13±0.13b | 2.37±0.13b |
|  | MK | 2.72±0.11a | 2.79±0.17a | 3.57±0.11a | 2.72±0.11a |
|  | HK | 1.98±0.09c | 2.16±0.13c | 2.62±0.11c | 2.02±0.07c |
| Alkaline phosphatase  (U·mg^-1^) | CK | 9.49±0.28d | 11.51±0.92d | 12.30±0.95d | 10.72±0.37d |
|  | LK | 15.32±1.3b | 18.51±0.56b | 21.22±1.09b | 17.12±0.77b |
|  | MK | 20.93±1.28a | 21.68±0.53a | 21.96±1.38a | 20.93±0.97a |
|  | HK | 11.39±0.54c | 12.23±0.55c | 13.35±0.50c | 12.18±0.60c |

Data are presented as mean ± standard error of the mean. Small letter in the same column means significant difference at *p* < 0.05. CK: potassium fertilizer application rate was 0 kg·ha^-1^; LK: potassium fertilizer application rate was 15 kg·ha^-1^; MK: potassium fertilizer application rate was 30 kg·ha^-1^; HK: potassium fertilizer application rate was 45 kg·ha^-1^.

**Supplementary data in 2021**

**TABLE S8** **Effects of different potassium fertilizer application rates on agronomic traits of thin-shelled Tartary buckwheat**

| Treatment | Plant  height (cm) | Node number  of main stem | Branch number of  main stem | Leaf number |
| --- | --- | --- | --- | --- |
| CK | 59.40±1.19d | 12.32±0.48c | 5.62±0.14d | 13.98±0.44d |
| LK | 73.37±1.32b | 14.15±0.32b | 7.24±0.12b | 21.09±0.56b |
| MK | 81.53±1.41a | 17.89±0.53a | 8.68±0.16a | 34.35±0.58a |
| HK | 67.62±1.88c | 12.93±0.57c | 6.37±0.26c | 16.42±0.33c |

Data are presented as mean ± standard error of the mean. Small letter in the same column means significant difference at *p* < 0.05. CK: potassium fertilizer application rate was 0 kg·ha^-1^; LK: potassium fertilizer application rate was 15 kg·ha^-1^; MK: potassium fertilizer application rate was 30 kg·ha^-1^; HK: potassium fertilizer application rate was 45 kg·ha^-1^.

**TABLE S9 Effects of different potassium fertilizer application rates on yield of thin-shelled Tartary buckwheat**

| Treatment | Grain number  per plant | Grain weight  per plant (g) | 100-grain weight  (g) | Yield  (kg·ha^-1^) |
| --- | --- | --- | --- | --- |
| CK | 206.2±7.7d | 2.41±0.05d | 1.18±0.03d | 743.9±21.39d |
| LK | 282.0±6.8b | 2.67±0.08b | 1.37±0.05b | 894.1±23.85b |
| MK | 304.6±4.4a | 2.84±0.08a | 1.62±0.04a | 1026.2±26.06a |
| HK | 248.4±9.7c | 2.52±0.06c | 1.25±0.04c | 810.6±26.93c |

Data are presented as mean ± standard error of the mean. Small letter in the same column means significant difference at *p* < 0.05. CK: potassium fertilizer application rate was 0 kg·ha^-1^; LK: potassium fertilizer application rate was 15 kg·ha^-1^; MK: potassium fertilizer application rate was 30 kg·ha^-1^; HK: potassium fertilizer application rate was 45 kg·ha^-1^.

**TABLE S10** **Effects of different potassium fertilizer application rates on gain weight (g) and grain-filling rate of thin-shelled Tartary buckwheat**

| Treatment | [Period](javascript:;) | | | | | G _max._  g (100·grain·day)^−1^ | G _mean_  g (100·grain· day)^−1^ |
| --- | --- | --- | --- | --- | --- | --- | --- |
|  | 7d | 14d | 21d | 28d | 35d |  |  |
| CK | 0.22±0.02d | 0.43±0.02d | 0.82±0.03d | 1.09±0.06d | 1.19±0.06d | 0.54±0.02d | 0.43±0.02d |
| LK | 0.32±0.01b | 0.58±0.01b | 1.06±0.03b | 1.31±0.06b | 1.38±0.06b | 0.82±0.04b | 0.71±0.03b |
| MK | 0.39±0.02a | 0.65±0.01a | 1.24±0.04a | 1.42±0.04a | 1.61±0.05a | 0.95±0.02a | 0.83±0.03a |
| HK | 0.28±0.02c | 0.50±0.03c | 0.90±0.04c | 1.23±0.03c | 1.26±0.05c | 0.71±0.03c | 0.52±0.03c |

Data are presented as mean ± standard error of the mean. Small letter in the same column means significant difference at p < 0.05. CK: potassium fertilizer application rate was 0 kg·ha^-1^; LK: potassium fertilizer application rate was 15 kg·ha^-1^; MK: potassium fertilizer application rate was 30 kg·ha^-1^; HK: potassium fertilizer application rate was 45 kg·ha^-1^. G_max:_ the maximum grain-filling rate; G_mean_: the mean grain-filling rate.

**TABLE S11 Effects of different potassium fertilizer application rates on starch synthase in grains of thin-shelled Tartary buckwheat**

| Index | Treatment | Period | | | | |
| --- | --- | --- | --- | --- | --- | --- |
|  |  | 7d | 14d | 21d | 28d | 35d |
| Soluble starch synthase  (SSS, U· mg^-1^·min^−1^) | CK | 13.14±0.12d | 14.82±0.11d | 18.74±0.23d | 17.18±0.23d | 16.78±0.66d |
|  | LK | 19.23±0.18b | 22.93±0.23b | 26.42±0.24b | 22.34±0.18b | 20.69±0.36b |
|  | MK | 26.14±0.33a | 31.54±0.45a | 35.27±0.12a | 26.83±0.76a | 22.41±0.24a |
|  | HK | 17.52±0.28c | 19.32±0.28bc | 23.68±0.39c | 19.59±0.22c | 18.62±0.23c |
| ADP-glucose pyrophosphorylase ( AGPase, U·g^-1^·min^−1^) | CK | 10.02±0.41d | 11.45±0.31d | 14.26±0.44d | 11.23±0.54d | 9.13±0.19d |
|  | LK | 18.23±0.24b | 21.42±0.26b | 22.68±0.18b | 19.12±0.27b | 16.95±0.24b |
|  | MK | 22.59±0.13a | 24.63±0.37a | 26.31±0.33a | 22.37±0.39a | 19.52±0.26a |
|  | HK | 14.17±0.22c | 15.86±0.45c | 18.03±0.42c | 16.98±0.33c | 13.10±0.23c |

Data are presented as mean ± standard error of the mean. Small letter in the same column means significant difference at *p* < 0.05. CK: potassium fertilizer application rate was 0 kg·ha^-1^; LK: potassium fertilizer application rate was 15 kg·ha^-1^; MK: potassium fertilizer application rate was 30 kg·ha^-1^; HK: potassium fertilizer application rate was 45 kg·ha^-1^.

**TABLE S12** **Effects of different potassium fertilizer application rates on antioxidant enzyme activity and MDA content of thin-shelled Tartary buckwheat**

| Index | Treatment | Period | | | |
| --- | --- | --- | --- | --- | --- |
|  |  | Seedling period | Flowering period | Grain-filling period | Maturity period |
| Superoxide dismutase  ( SOD, U·g^-1^·h^-1^ ) | CK | 156.24±7.64d | 179.11±9.79d | 203.15±8.92d | 133.28±7.87d |
|  | LK | 240.31±9.11b | 281.63±8.09b | 299.71±13.36b | 218.03±14.36b |
|  | MK | 349.57±8.45a | 352.05±9.51a | 403.18±8.97a | 324.21±12.93a |
|  | HK | 201.92±9.68c | 241.84±6.57c | 259.82±10.45c | 206.39±7.82c |
| Peroxidase  (POD,U·g^-1^·h^-1^) | CK | 173.15±4.61d | 210.88±8.54d | 243.27±6.76d | 195.68±9.19d |
|  | LK | 244.98±9.77b | 271.25±9.88b | 291.23±8.55b | 252.13±10.85b |
|  | MK | 301.36±5.79a | 321.97±8.39a | 350.16±7.97a | 342.93±8.82a |
|  | HK | 195.61±5.97c | 242.39±7.86c | 274.92±9.44c | 223.18±6.94c |
| Malonaldehyde (MDA, μmol·g^−1^) | CK | 1.89±0.06a | 2.05±0.09a | 3.96±0.04a | 5.17±0.06a |
|  | LK | 1.76±0.04b | 1.91±0.09b | 2.82±0.06c | 4.23±0.04c |
|  | MK | 1.61±0.05c | 1.76±0.05c | 2.73±0.06d | 3.30±0.06d |
|  | HK | 1.85±0.04a | 1.97±0.05b | 3.20±0.05b | 4.72±0.07b |

Data are presented as mean ± standard error of the mean. Small letter in the same column means significant difference at *p* < 0.05. CK: potassium fertilizer application rate was 0 kg·ha^-1^; LK: potassium fertilizer application rate was 15 kg·ha^-1^; MK: potassium fertilizer application rate was 30 kg·ha^-1^; HK: potassium fertilizer application rate was 45 kg·ha^-1^.

**TABLE S13 Effects of different potassium fertilizer application rates on root morphology and root activity of thin-shelled Tartary buckwheat**

| Index | Treatment | Period | | | |
| --- | --- | --- | --- | --- | --- |
|  |  | Seedling period | Flowering period | Grain-filling period | Maturity period |
| Root activity  (μg·g·h^-1^) | CK | 52.02±1.49d | 43.81±2.53d | 33.12±0.83d | 21.32±0.46d |
|  | LK | 75.79±2.58b | 63.35±2.56b | 56.21±0.84b | 32.61±0.67b |
|  | MK | 98.31±3.83a | 78.24±1.87a | 64.38±0.46a | 36.38±0.79a |
|  | HK | 68.62±2.37c | 52.02±1.90c | 41.52±0.96c | 25.38±0.51c |
| Root length  (cm) | CK | 16.42±0.40d | 18.13±1.15d | 19.42±1.07d | 21.09±0.77d |
|  | LK | 32.91±0.78b | 36.94±2.26b | 38.64±0.71b | 40.48±0.56b |
|  | MK | 44.82±0.51a | 44.58±2.23a | 48.21±0.96a | 55.27±0.80a |
|  | HK | 22.54±0.81c | 31.90±1.63c | 33.34±0.86c | 36.10±0.76c |
| Root surface area  (cm^2^ ) | CK | 4.07±0.11d | 4.32±0.12d | 5.27±0.15d | 5.68±0.26d |
|  | LK | 5.95±0.10b | 6.48±0.16b | 11.16±0.58b | 12.04±0.69b |
|  | MK | 6.42±0.12a | 12.42±0.53a | 13.08±0.31a | 14.24±0.29a |
|  | HK | 5.02±0.13c | 5.96±0.28c | 7.64±0.13c | 7.92±0.33c |
| Root volume  (cm^3^) | CK | 0.14±0.01c | 0.18±0.01c | 0.46±0.01d | 0.77±0.02d |
|  | LK | 0.19±0.01b | 0.34±0.03b | 0.83±0.02b | 1.42±0.04b |
|  | MK | 0.23±0.03a | 0.71±0.03a | 1.12±0.04a | 1.93±0.07a |
|  | HK | 0.15±0.02c | 0.19±0.01c | 0.59±0.02c | 1.10±0.05c |

Data are presented as mean ± standard error of the mean. Small letter in the same column means significant difference at *p* < 0.05. CK: potassium fertilizer application rate was 0 kg·ha^-1^; LK: potassium fertilizer application rate was 15 kg·ha^-1^; MK: potassium fertilizer application rate was 30 kg·ha^-1^; HK: potassium fertilizer application rate was 45 kg·ha^-1^.

**TABLE S14** **Effects of different potassium fertilizer application rates on available nutrients and soil enzyme activities in rhizosphere soil**

| Index | Treatment | Period | | | |
| --- | --- | --- | --- | --- | --- |
|  |  | Seedling period | Flowering period | Grain-filling period | Maturity period |
| Available nitrogen  (mg·kg^-1^) | CK | 8.61±0.11c | 9.23±0.11d | 15.82±0.56c | 8.22±0.31d |
|  | LK | 9.92±0.14b | 13.42±0.40b | 18.79±0.58b | 11.37±0.52b |
|  | MK | 14.24±0.68a | 16.56±0.44a | 21.06±0.51a | 13.69±0.71a |
|  | HK | 9.89±0.28b | 11.34±0.61c | 18.10±0.56b | 9.94±0.69c |
| Available phosphorus  (mg·kg^-1^) | CK | 10.43±0.31d | 16.05±0.63d | 32.05±0.40d | 12.06±0.69d |
|  | LK | 15.96±0.61b | 24.09±0.74b | 39.11±0.44b | 26.95±0.67b |
|  | MK | 28.34±0.63a | 30.16±0.49a | 46.28±0.72a | 32.41±0.72a |
|  | HK | 12.79±0.56c | 17.95±0.83c | 34.92±0.71c | 15.93±0.71c |
| Available potassium  (mg·kg^-1^) | CK | 19.09±0.40d | 32.88±0.81d | 38.76±0.87d | 17.93±0.61d |
|  | LK | 24.92±0.95c | 35.20±0.58c | 41.29±0.98c | 23.30±0.88c |
|  | MK | 40.14±0.79b | 45.37±0.75b | 50.63±0.89b | 31.84±0.79b |
|  | HK | 47.66±0.63a | 56.94±0.97a | 59.65±0.79a | 46.73±0.86a |
| Organic matter  (g·kg^-1^) | CK | 25.88±0.53d | 29.26±0.74c | 36.16±0.82c | 22.13±0.76d |
|  | LK | 33.04±0.97b | 36.84±0.62b | 42.48±0.86b | 35.02±0.44b |
|  | MK | 40.57±0.83a | 42.91±0.53a | 47.36±0.53a | 38.95±0.72a |
|  | HK | 30.05±0.64c | 35.12±0.45b | 41.02±0.98b | 31.14±0.83c |
| Urease enzyme  mg (g·d)^-1^ | CK | 1.69±0.08d | 2.08±0.12d | 2.44±0.13d | 1.83±0.07d |
|  | LK | 2.43±0.08b | 2.55±0.12b | 3.22±0.16b | 2.43±0.08b |
|  | MK | 2.82±0.09a | 3.16±0.14a | 3.80±0.12a | 2.80±0.15a |
|  | HK | 2.05±0.13c | 2.34±0.10c | 2.86±0.15c | 2.12±0.11c |
| Alkaline phosphatase  (U·mg^-1^) | CK | 9.62±0.14d | 11.97±0.89d | 13.99±0.73c | 11.10±0.52d |
|  | LK | 16.95±0.99b | 19.15±0.53b | 22.09±0.57b | 17.49±0.92b |
|  | MK | 21.78±0.91a | 22.05±1.10a | 22.95±1.27a | 21.33±0.95a |
|  | HK | 11.71±0.73c | 12.78±0.80c | 13.91±0.53c | 12.49±0.80c |

Data are presented as mean ± standard error of the mean. Small letter in the same column means significant difference at *p* < 0.05. CK: potassium fertilizer application rate was 0 kg·ha^-1^; LK: potassium fertilizer application rate was 15 kg·ha^-1^; MK: potassium fertilizer application rate was 30 kg·ha^-1^; HK: potassium fertilizer application rate was 45 kg·ha^-1^.
